# Advances in Germplasm Resources, Bioactivities, and Comprehensive Utilization of Canistel (*Pouteria campechiana* (Kunth) Baehni)

**DOI:** 10.3390/plants15203230

**Published:** 2026-10-21

**Authors:** Wen-Yan Li, Cai-Xia Zhou, Zhi-Luo Zhou, Mei-Ying Lu, Zhen-Ling Yan, Pei-Si Luo, Jing Zhou, You Wei

**Affiliations:** 1 Guangxi South Subtropical Agricultural Sciences Research Institute, Nanning 530007, China; 2 Guangxi Innovation Center for Fruit and Vegetable Industry Technology, Nanning 530007, China; 3 Chongzuo Key Laboralory of Crop Gene Resources and Genctic, Chongzuo 532415, China

**Keywords:** yellow sapote, egg fruit, phytochemistry, carotenoids, antioxidant activity, functional food, underutilized crop

## Abstract

Canistel (*Pouteria campechiana* (Kunth) Baehni) is a tropical evergreen fruit tree native to Mesoamerica, with expanding cultivation in southern China and other tropical regions. This review provides a comprehensive and critical synthesis of current knowledge on canistel, covering botanical characteristics, germplasm resources and genetic diversity, propagation and cultivation techniques, nutritional composition, phytochemical diversity, pharmacological bioactivities, and food processing applications. In its center of origin, germplasm conservation is threatened by genetic erosion, while Chinese breeding programs have released improved cultivars such as ‘Xiantao 1’, ‘Xiantao 2’, and ‘Yunre-205’. Propagation methods, including seed germination and grafting, have been optimized, and the species exhibits broad stress tolerance. The fruit is rich in carotenoids (especially lycopene and β-carotene), phenolic compounds, and essential minerals, and its seeds contain high-amylose starch (up to 68.1%) with resistant starch properties. Preclinical studies demonstrate antioxidant, anti-inflammatory, hepatoprotective, analgesic, and immunomodulatory activities, although clinical and long-term toxicological data are lacking. Advances in carotenoid encapsulation and oil-based fortification offer promising routes for functional food development. To realize its full potential, coordinated international efforts are needed to broaden genetic resources, elucidate metabolic pathways, develop scalable processing technologies, and validate health benefits through rigorous clinical trials.

## 1. Introduction

Canistel (*Pouteria campechiana* (Kunth) Baehni, synonym *Lucuma nervosa* A. DC.), is an evergreen fruit tree belonging to the family Sapotaceae, native to the lowlands of central America and southern Mexico [1,2]. The tree typically reaches 8–20 m in height, with a trunk diameter of 25–60 cm, and produces distinctive berry fruits that vary from round to ovoid or spindle-shaped, often with a pointed apex. When ripe, the fruit exhibits a pale orange-yellow to golden-yellow rind, while the flesh is soft, dry, and mealy, with a color and texture reminiscent of cooked egg yolk—hence its common name “egg fruit” or “yellow sapote” [3,4,5].

The fruit is valued for its distinctive sweet flavor and high nutritional density. It can be consumed fresh or processed into jams, creams, beverages, and fruit wines [6]. Beyond its nutritional value, various parts of the plant—including leaves, seeds, and bark—contain bioactive secondary metabolites with demonstrated antioxidant, antibacterial, and anti-inflammatory properties, and have been used in traditional Mayan and Southeast Asian medicine to treat inflammation, ulcers, fever, and skin rashes [7,8,9].

A recent review provided a broad overview of the phytochemical and nutraceutical aspects of multiple Sapotaceae species native to Mesoamerica, including canistel, mamey sapote (*Pouteria sapota*), green sapote (*Pouteria viridis*), and sapodilla (*Manilkara zapota*) [10]. While that work offers valuable contextual information on the family-level diversity of bioactive compounds, it does not provide a systematic, species-specific synthesis of canistel, particularly regarding its germplasm resources, cultivation physiology, postharvest management, and value-added processing applications. These aspects are critical for translating canistel from a locally consumed fruit into a commercially viable crop with sustained supply chains and diversified product portfolios.

In China, the tropical specialty fruit industry has expanded rapidly over the past two decades, drawing increasing attention to canistel as a high-value rare fruit with considerable commercial potential [11,12]. In recent years, several agricultural research institutions in China have developed several excellent varieties of canistel. Nevertheless, research on canistel remains nascent compared to major tropical fruits such as mango or avocado, and a systematic, critical synthesis of existing knowledge is lacking.

This review critically examines the current state of canistel research through the lens of three overarching questions: (1) To what extent have germplasm resources been characterized and utilized for breeding? (2) What is the evidence base for the purported health benefits, and how robust are the pharmacological data? (3) What technical and knowledge gaps impede the translation of canistel from a locally consumed fruit to a globally recognized functional food ingredient? Rather than providing a comprehensive but uncritical compilation of all the available literature, we focus on identifying the methodological strengths and weaknesses, the unresolved contradictions, and the most impactful future directions. We cover botanical characteristics, germplasm resources and genetic diversity, cultivation and propagation techniques, nutritional composition and phytochemical profiles, pharmacological bioactivities, and food processing applications—but at each stage, we interrogate the quality and consistency of the evidence rather than simply cataloging it.

## 2. Botanical Characteristics

Canistel is a medium-sized, erect evergreen tree, typically attaining 8–20 m in height, with a trunk diameter of 25–60 cm. The entire plant is rich in white, gummy latex, a characteristic feature of many Sapotaceae species [1,2]. The leaves are alternate, clustered in a pseudowhorl at the branch tips, elliptic to oblanceolate or obovate, and measuring 6–25 cm in length and 2.5–8 cm in width. The lamina is bright green and glabrous, with entire margins and prominent reticulate venation [13].

The inflorescences consist of small, fragrant, greenish-white flowers, approximately 1 cm in diameter. Flowers are solitary or clustered in leaf axils or on leafless nodes, with pedicels 5–12 mm long. The flowers are hermaphroditic, bearing numerous stamens and a single superior ovary [14]. Pollination is primarily entomophilous, though self-compatibility has been reported in some cultivars.

The fruit is a berry, highly variable in shape (round, ovoid, subglobose, or spindle-shaped), often with a characteristic pointed apex. Fruit dimensions range from 7 to 12.5 cm in length and 4 to 7.5 cm in width. Immature fruits are green, hard, and rich in latex, while mature fruits develop a pale orange-yellow to golden-yellow rind. The flesh becomes soft, dry, and mealy upon ripening, emitting a subtle musky aroma. The pulp is rich in carbohydrates, proteins, vitamins (C, A, and B-complex), dietary fiber, various fatty acids, flavonoids, polyphenols and abundant minerals including phosphorus, iron, and calcium [4,15,16,17].

Each fruit typically contains 1–5 seeds, which are ovoid to globose, 2–5.3 cm long and 1.25–3.2 cm wide, with a chestnut-brown, glossy testa. The ventral side is curved and marked with a pale brown to greyish-white hilum.

Seedling trees commence flowering and fruiting at 4–5 years of age, with approximately 5–6 months elapsing from anthesis to fruit maturity [18]. The ripening period is geographically variable: August–October in Mexico, July and December in China, and May and September in Vietnam [1,14]. The species thrives under tropical and subtropical conditions with ample rainfall, at elevations below 1800 m, and exhibits tolerance to a range of soil types provided drainage is adequate [19].

## 3. Germplasm Resources and Cultivar Improvement

### 3.1. Germplasm Collection and Evaluation

Conservation of canistel germplasm is gaining global attention, especially in its native range. In Mexico’s Yucatán Peninsula, likely the center of domestication, the species—locally called kanisté—was traditionally grown in Mayan home gardens, but surveys now show a sharp decline: only 9 of 200 orchards still retain trees, raising concerns about genetic erosion. A genetic assessment of 40 wild and cultivated trees using SSR and ISSR markers found moderate species-level diversity (SSR: *H*_E_ = 0.52 ± 0.031), with natural habitats (SSR: *H*_E_ = 0.64 ± 0.032) being more diverse than anthropogenic ones (SSR: *H*_E_ = 0.39 ± 0.058). Most SSR loci (16 of 18) were linked to protein-coding regions involved in sugar transport, seed development, floral scent, and disease resistance, offering tools for marker-assisted conservation and breeding [20].

In China, systematic collection of canistel germplasm began only in the mid-1990s. Since 1995, the researchers in Guangxi South Subtropical Agricultural Sciences Research Institute have conducted extensive collection, preservation, and evaluation efforts, establishing a 30 mu (approximately 2 hectare) germplasm nursery containing 85 accessions. This program has screened several superior genotypes from seedling populations and propagated them clonally [21,22]. The superior variety “Yunre-205” was selected by the Tropical and Subtropical Economic Crops Institute of the Yunnan Academy of Agricultural Sciences. This variety features large fruits, high yield, and excellent quality [23].

### 3.2. Improved Cultivars

Canistel cultivars can be broadly categorized by flesh texture into three types: moist-fleshed types (juicy and creamy, preferred for fresh consumption), dry-fleshed types (mealy and dense, ideal for processing), and medium-dry types (intermediate texture, suitable for both fresh and processed uses). This classification is critical for end-use selection but has received insufficient attention in breeding programs.

Internationally, several canistel cultivars have been developed outside China. In South Florida, the Fairchild Tropical Botanic Garden maintains a collection of 12 superior cultivars, among which ‘Bruce’, ‘Fairchild 2’, ‘Keisau’, ‘Oro’, and ‘Trompo’ have been evaluated for cottage industry applications. ‘Bruce’ is characterized by large fruit (averaging 375 g) with moist flesh and high yield capacity. ‘Oro’ and ‘Trompo’ produce slightly moist, very sweet flesh with good flavor, and ‘TREC 9680’ is notable for its attractive size, shape, and color. In Mexico, native canistel populations exhibit substantial morphological variability in fruit weight (180–330 g), shape (subglobose to ovoid), number of seeds (1–5), and soluble solid content (up to 33%) across different geographical zones [24]. Mexican selections are typically dry-fleshed with intense yellow-orange pulp color, characteristics favored for processing into flours and purees. In Cuba and the Caribbean, locally adapted landraces are known for their sweet, moist flesh and are consumed primarily as fresh dessert fruits [25].

Three improved cultivars have been formally released in China to date. ‘Xiantao 1’—Bred by the Guangxi South Subtropical Agricultural Sciences Research Institute, this cultivar exhibits vigorous tree growth with a spreading canopy. The leaves are oblanceolate, and flowers are greenish-white. The peach-shaped fruits have an average weight of 233.7 g, with a thin, easily removable golden-yellow peel. The flesh is egg-yolk-yellow, mealy, sweet, and aromatic, containing approximately three seeds per fruit. The edible rate reaches 70.1%, with total sugar of 27.8%, vitamin C of 0.64 mg/g, starch of 3.6%, and protein of 1.42%. Storage tolerance is approximately 12 days at ambient conditions. In Guangxi, trees begin bearing in the second year after planting; flowering occurs in mid-to-late April, with peak ripening from late December to early January. At five years of age, average yield is 12.9 kg per tree, reaching approximately 150 kg at full production. The cultivar shows strong resistance to environmental stresses and pests [26] (Figure 1). ‘Xiantao 2’—Also bred by the Guangxi South Subtropical Agricultural Sciences Research Institute, this cultivar has a round-headed canopy, oblanceolate leaves, and hermaphroditic flowers. Fruits are peach-shaped with an average weight of 158.54 g (maximum 284.72 g). The peel is greenish-yellow, thin, and smooth; flesh is egg-yolk-yellow, containing 1–2 seeds. In suitable cultivation areas of Guangxi and Guangdong, the main fruiting branches are spring shoots. Flowering occurs from mid-April to early May (approximately 100 days), with peak ripening from late January to early February of the following year—a fruit development period of about 210 days. The harvest window lasts approximately 50 days, with relatively concentrated ripening [27] (Figure 2). ‘Yunre-205’—For this cultivar, tree vigor is moderate; the canopy is round-headed, and the trunk is grayish white. The leaves are oblanceolate, with a mean leaf length of 24.14 cm, a mean width of 5.86 cm, and a leaf shape index of 4.12. The leaf blades are undulate and fold inward on both sides. Newly emerged unexpanded leaves are brown and covered with brown pubescence, whereas mature leaves are dark green, leathery, glossy, and glabrous on both surfaces. The flowers are medium-sized, bisexual, and campanulate, with a mean corolla diameter of 6.5 mm; they are clustered or solitary, pale yellow, and covered with short brown pubescence. The fruit is peach-shaped. Immature fruits are green, and the fruit surface is densely covered with brown pubescence. The mature pericarp is orange-yellow, with a mean longitudinal diameter of 13.5 cm, a transverse diameter of 5.8 cm, and a single-fruit weight of 210.7 g. The pedicel end is slightly depressed, and the fruit apex has a nipple-like protrusion. The flesh is yellow, soft in texture, and sweet and delicate in taste. The seeds are elliptic; the seed coat is reddish brown, hard, and glossy. The mean number of seeds per fruit is 2–3, and the mean seed weight is 15.4 g [23,28,29] (Figure 3). The major economic traits of representative canistel cultivars from China and abroad are shown in Table 1.

### 3.3. Molecular Markers and Genetic Diversity

Molecular marker technologies have been increasingly applied to canistel germplasm identification and genetic diversity analysis. Sun and Wang [30] developed a modified CTAB protocol for genomic DNA extraction that effectively removes abundant polysaccharides and proteins from canistel tissues, achieving high-purity DNA (74% of samples with A_260_/A_280_ ratios of 1.6–1.8) and an average yield of 444 μg/g—sufficient for PCR-based applications.

Xing and Wang [31] optimized the ISSR-PCR reaction system for canistel. More recently, Zhou et al. [32] employed an L_16_(4^5^) orthogonal design to establish an optimized SCoT-PCR reaction system (20 μL): 80 ng template DNA, 3 mmol/L Mg^2+^, 0.25 mmol/L dNTPs, 0.4 μmol/L primers, and 2 U Taq polymerase. Four core SCoT primers (SCoT39, SCoT40, SCoT41, and SCoT42) were screened, enabling the construction of a preliminary DNA fingerprinting profile for canistel.

At the genomic level, Jo et al. [33] reported the first complete chloroplast genome sequence of canistel, revealing a circular genome of 157,922 bp, comprising a large single-copy (LSC) region of 87,122 bp, a small single-copy (SSC) region of 18,559 bp, and two inverted repeat (IR) regions of 26,120 bp each. The overall A-T content is 63.2%. The chloroplast genome contains 113 genes (79 protein-coding, 30 tRNA, and 4 rRNA genes) with 91 simple sequence repeat (SSR) loci identified. Niu et al. [34] independently characterized the chloroplast genome (157,920 bp, 113 genes) with highly similar structural features. Phylogenetic analyses consistently place canistel within a well-supported clade alongside other Sapotaceae species [35]. Peña-Ramírez et al. [36] further resolved the complete nuclear genome sequence of a wild Mexican accession using Illumina Hi-Seq paired-end sequencing (2 × 150 bp), providing a foundational genomic resource for future molecular breeding.

In the center of origin, recent studies have advanced our understanding of canistel genetic diversity. Jiménez-Parra et al. [20] analyzed 40 wild and cultivated kanisté trees from the Yucatán Peninsula using 18 SSR markers and 167 ISSR loci, revealing two main genetic groups with substructures related to geographical origin. Genetic diversity was higher in natural habitats (SSR: *H*_E_ = 0.64) than in anthropogenic environments (SSR: *H*_E_ = 0.39), suggesting that cultivated populations have experienced genetic erosion. NCBI BLAST analysis further identified SSR loci associated with protein-coding regions involved in sugar transport (SWEET family), seed development, floral scent (geranyl linalool synthase), and disease immunity (MAPKKK NPK1-like), providing candidate markers for future breeding. A separate morphological characterization study in Mexico collected semi-ripe fruits from three geographically neighboring zones and identified significant variation in fruit weight (180–330 g), pulp weight (198.88 g maximum), number of seeds, and total soluble solids, with leaf area, perimeter, and minor diameter as the main discriminatory variables [25]. These findings establish a foundation for selecting outstanding Mexican genotypes for large-scale propagation and for future association mapping studies.

## 4. Cultivation and Propagation Techniques

### 4.1. Propagation Methods

Canistel is propagated through two primary methods: seed propagation and grafting. Seed propagation—Canistel seeds lack dormancy and can be sown directly. Li et al. [37] investigated the effects of gibberellic acid (GA_3_) on germination, finding that low concentrations promote germination and seedling growth, whereas high concentrations are inhibitory. The optimal concentration (100 mg/L) achieved a germination rate of 92.53%, germination potential of 57.80%, and maximal radicle and plumule elongation. Grafting—Grafting is essential for maintaining cultivar traits and accelerating fruiting. Zhou et al. [38] systematically evaluated the effects of scion type, grafting method, and season using ‘Xiantao 1’ as scion material. The results demonstrated the following: (1) lignified branch scions achieved the highest survival rate (99.33%), significantly outperforming semi-lignified branches (80.00%) and terminal buds (58.67%); (2) cleft grafting achieved the highest survival rate (98.00%), surpassing cut grafting (80.00%) and splice grafting (61.33%); and (3) spring grafting (March–April) achieved the highest survival rate of 100%, followed by autumn (85.33%) and winter (55.33%). Thus, cleft grafting using lignified branches in spring is strongly recommended. Zhang et al. [39] similarly reported that multiple-bud cleft grafting in February achieved the highest survival rate in the Nujiang dry–hot valley region of Yunnan.

Outside China, propagation research has focused on seed germination optimization and root system development. Granados-Vega et al. [40] systematically reviewed ex vitro propagation strategies for *P. campechiana*, identifying manual scarification and proper seed orientation as critical factors for promoting radicle and plumule development. Root pruning of malformed taproots before transplanting significantly improved plant development and enhanced natural association with arbuscular mycorrhizal fungi, leading to higher-quality seedling production. In Florida, canistel grafting onto seedling rootstocks is commonly practiced to maintain cultivar traits, though comprehensive studies on optimal rootstock–scion combinations remain limited [24].

### 4.2. Orchard Management

Canistel exhibits broad adaptability, tolerating poor soil fertility, moderate drought, and cold conditions, with no obvious chilling injury observed at extreme minimum temperatures above 2.5 °C [7]. Mature bearing trees require three annual fertilizer applications: flower-promoting fertilizer (late March), fruit-enlarging fertilizer (early July), and post-harvest fertilizer (early February). Young trees should be allowed to prioritize vegetative growth during the first three years to establish a robust canopy structure, while mature trees benefit from heavy pruning after harvest to rejuvenate fruiting branches [23]. Regarding bagging technology, Lu et al. [41] studied the effects of fruit bagging on ‘Xiantao 1’, finding that bagging advanced fruit maturation by approximately 10 days. Double-layer paper bags with black inner and brown outer linings resulted in the earliest and most concentrated ripening. Bagging also significantly improved skin smoothness and promoted uniform coloration, though it reduced total sugar, vitamin C, and starch contents—a trade-off that warrants consideration based on market preferences.

### 4.3. Stress Tolerance

Canistel demonstrates notable stress resistance. During the severe prolonged cold event in southern China during the winter of 2007–2008, when extreme minimum temperatures in Longzhou County, Guangxi, reached 2.0 °C, most accessions showed no visible injury aside from purple-red discoloration of old leaves [21]. During the severe drought of 2009–2010 in southwestern Guangxi, canistel maintained normal growth and development under prolonged non-irrigated conditions [7,42,43]. The species also exhibits strong resistance to pests and diseases, with only minor occurrences of sooty mold, leaf beetles, and flatid planthoppers, requiring minimal pesticide intervention throughout the year [44].

## 5. Nutritional Composition and Phytochemistry

### 5.1. Proximate Nutritional Profile

Canistel fruit is characterized by a dense nutritional composition. The edible portion accounts for over 70% of fruit weight, with soluble sugar exceeding 14%, reducing sugar being above 10%, starch ranging from 5.6% to 8.1%, and crude fat being between 1.0% and 1.14%. Vitamin C content is 24.3 mg per 100 g fresh pulp [44]. The fruit is also a good source of phosphorus, iron, calcium, carotenoids, and essential amino acids [6]. Importantly, canistel fruit does not accumulate hazardous heavy metals (lead, cadmium, chromium), confirming its safety for human consumption. The seeds, often discarded as processing waste, contain up to 13.89 g/100 g starch, along with considerable crude protein, flavonoids, polyphenols, and polysaccharides [3,45]. The seed starch has attracted particular research interest due to its unique properties (see Section 7.1). The peel comprises 8.9% protein, 17.3% fiber, and 10.7% lipids and contains substantial phenolic compounds, though it is currently underutilized [3]. The optimal harvest stage is defined as the period when the fruit skin transitions from green to yellowish-green.

### 5.2. Phenolic Compounds

Canistel is rich in phenolic secondary metabolites with notable antioxidant potential. Using SIM LC-MS, Ma et al. [46] identified four polyphenolic antioxidants from canistel fruit: gallic acid (16.85 ppm), (+)-gallocatechin (5.62 ppm), (+)-catechin (1.04 ppm), and myricitrin (2.82 ppm). Kubola et al. [47] identified two phenolic acids (hydroxybenzoic acid and hydroxycinnamic acid) and six flavonoids (rutin, myricetin, luteolin, quercetin, apigenin, and kaempferol) from wild Thai canistel fruit.

Hernandez et al. [48] isolated and characterized six stilbenoid dimers and tetramers (ampelopsin B, balanocarpol, ε-viniferin diol, ampelopsin A, hopeaphenol, and vaticaphenol A) alongside six flavonoid glycosides (taxifolin 3-O-α-arabinofuranoside, trans-taxifolin 3-O-α-arabinopyranoside, quercetin 3-O-α-rhamnopyranoside, myricetin 3-O-α-rhamnopyranoside, taxifolin 3-O-α-rhamnopyranoside, and quercetin 3-O-β-arabinopyranoside) from canistel leaves. Elsayed et al. [49] further identified protocatechuic acid, gallic acid, quercetin, and myricetin from both leaves and seeds.

Total phenolic and flavonoid contents vary considerably across tissues [50,51]. Leaves generally exhibit the highest levels, followed by seeds, while peel and pulp contain lower amounts [52,53,54,55,56]. This differential distribution has important implications for selecting appropriate plant parts for specific functional applications.

### 5.3. Carotenoids

Canistel is a particularly rich source of carotenoids, which account for its characteristic yellow-orange pigmentation. Costa et al. [57] reported total carotenoid content of 226 ± 4 μg/g in canistel, predominantly violaxanthin and neoxanthin. de Lanerolle et al. [58] found a much wider range (1.87 to 23.47 mg/g DW) across Sri Lankan accessions, suggesting substantial genotypic or environmental variation—a point meriting systematic investigation. Using deep eutectic solvents (DL-menthol combined with capric acid), Xiao [59] optimized carotenoid extraction, achieving total carotenoid content of 800.34 ± 1.34 μg/g DW. HPLC analysis identified lycopene as the most abundant carotenoid (498.66 ± 1.23 μg/mL DW), followed by β-carotene—a profile distinct from those of many other tropical fruits. This high lycopene content positions canistel as a promising natural source for lycopene extraction, with potential applications in the nutraceutical and food coloring industries.

A notable discrepancy exists in the reported carotenoid content of canistel across studies. This more than 30-fold difference likely reflects multiple confounding factors: genotypic variation, differences in extraction efficiency, distinct detection methods, and potentially different fruit maturity stages. Critically, none of the existing studies have systematically compared extraction methods on the same sample set, making it impossible to distinguish biological from methodological variation. Future work should prioritize method standardization and inter-laboratory validation to establish a reliable baseline for canistel carotenoid content.

### 5.4. Triterpenoids

Ren et al. [60] isolated and identified eight triterpenoids from canistel leaves, including two novel compounds—lucumic acid A and lucumic acid B. Ragasa et al. [61] additionally reported sterols and triterpenoids from the stem bark. Compared to the phenolic and carotenoid profiles, the triterpenoid composition of canistel has received minimal attention, with only two studies reporting structural characterization; their bioactivities and biosynthetic origins remain entirely unexplored. Given the established anti-inflammatory and cytotoxic properties of triterpenoids in other Sapotaceae species, this represents a substantial knowledge gap meriting targeted phytochemical and pharmacological investigation.

### 5.5. Volatile Compounds

The characteristic aroma of canistel is determined by its volatile fraction. Pino [62] identified 49 volatile compounds from mature fruit, with methyl 2-hydroxy-4-methylpentanoate and S-methyl 2-propenethioate as the major constituents. Ma et al. [45] identified 22 volatile compounds from seeds, predominantly oleic acid and hexadecanoic acid. The limited overlap between pulp and seed volatile profiles underscores the distinct biosynthetic pathways operating in different tissues.

## 6. Bioactivities and Pharmacological Effects

### 6.1. Antioxidant Activity

Substantial in vitro evidence demonstrates that canistel fruits, leaves, bark, and seeds possess significant antioxidant activity, closely correlated with their phenolic, flavonoid, and carotenoid contents [63,64]. Ma et al. [46] evaluated antioxidant capacity using the 2,2-diphenyl-1-picrylhydrazyl (DPPH) radical scavenging assay and found that the ethyl acetate fraction of canistel fruit exhibited an IC_50_ of 146.3 μg/mL, consistent with its phenolic content. Manthra et al. [65] reported that methanolic bark extracts showed high scavenging activity in multiple assays (2,2′-azino-bis(3-ethylbenzothiazoline-6-sulfonic acid) [ABTS] and DPPH).

Notably, Hidayah et al. [54] demonstrated that seed extracts exhibited the strongest antioxidant activity (IC_50_ = 0.5 μg/mL), followed by leaf (IC_50_ = 0.8 μg/mL), peel (IC_50_ = 8.0 μg/mL), and pulp (IC_50_ = 29.0 μg/mL)—a ranking consistent with their respective total phenolic and flavonoid contents. Using response surface methodology, Ma et al. [66] optimized ultrasound-assisted polyphenol extraction from pulp and seeds, finding that seed polyphenol yield (2.65%) substantially exceeded that of pulp (0.74%), with seed polyphenols exhibiting significantly stronger scavenging activities against DPPH, hydroxyl radicals, and superoxide anions. These findings highlight the potential of canistel seeds—currently a processing byproduct—as a high-value source of natural antioxidants.

### 6.2. Anti-Inflammatory and Immunomodulatory Activity

Several lines of evidence support the immunomodulatory and anti-inflammatory properties of canistel extracts. Chan-Zapata et al. [67] investigated the effects of methanolic leaf extract on murine peritoneal macrophages, finding that non-cytotoxic concentrations significantly stimulated phagocytic activity and promoted nitric oxide (NO), hydrogen peroxide, and cytokine production (tumor necrosis factor-α (TNF-α) and interleukin-6 (IL-6)), suggesting the potential to activate innate immune responses.

In a carrageenan-induced rat paw edema model, Elsayed et al. [49] reported that seed ethanolic extract exhibited up to 85% inhibition of inflammation, demonstrating superior efficacy compared to leaf extract. Luo [68] purified an acidic polysaccharide fraction (PCP-A) with a molecular weight of 1.58 × 10^3^ kDa from canistel and, using a dextran sulfate sodium (DSS)-induced mouse model of ulcerative colitis, demonstrated that PCP-A significantly reduced pro-inflammatory cytokines (TNF-α, IL-6, and IL-1β) in colon tissue while elevating anti-inflammatory IL-10. Mechanistically, these effects were associated with inhibition of the p38 mitogen-activated protein kinase (p38MAPK) and nuclear factor-κB (NF-κB) signaling pathways, alongside upregulation of peroxisome proliferator-activated receptor γ (PPAR-γ) expression.

Practical applications have also been explored: Corona et al. [69] found that dietary supplementation with canistel pulp nanocapsules enhanced non-specific defense mechanisms in laying hens, reducing infection-induced morbidity and mortality, suggesting potential veterinary nutraceutical applications.

### 6.3. Hepatoprotective Activity

Aseervatham et al. [70] evaluated the hepatoprotective effect of canistel aqueous extract in an acetaminophen (paracetamol)-induced acute liver injury rat model. Pre-treatment with 150 and 250 mg/kg extract reduced in a dose-dependent manner the elevated activities of serum liver injury markers—aspartate aminotransferase (AST), alanine aminotransferase (ALT), and alkaline phosphatase (ALP). Concurrently, hepatic antioxidant enzymes (superoxide dismutase and catalase) and non-enzymatic glutathione (GSH) levels were significantly restored, while malondialdehyde (MDA) content was reduced. Histopathological examination confirmed marked attenuation of hepatocellular necrosis and inflammatory infiltration. At 250 mg/kg, the hepatoprotective effect was comparable to that of the positive control silymarin (50 mg/kg).

### 6.4. Analgesic Activity

Déciga-Campos et al. [71] systematically investigated a traditional Maya herbal preparation (PCCA) composed of equal proportions of canistel, star apple (*Chrysophyllum cainito*), lemon (*Citrus limonum*), and soursop (*Annona muricata*). In the rat formalin test, oral PCCA (50–400 mg/kg) produced dose-dependent analgesia, with inhibition rates of 64.8% and 67.6% in phases I and II, respectively, at 400 mg/kg. In a capsaicin-induced pain model, local injection also demonstrated concentration-dependent antinociception, reducing pain responses by 69.5% at the maximum dose. Using the mouse hot-plate test, Elsayed et al. [49] confirmed that both leaf and seed ethanolic extracts (100 and 200 mg/kg) exhibited significant analgesic activity, with leaf extract showing superior efficacy.

### 6.5. Other Bioactivities

Additional pharmacological activities have been reported, albeit with varying levels of mechanistic depth: (1) Antidiabetic—Pai and Shenoy [72] demonstrated in vitro that methanolic leaf and fruit extracts effectively inhibit α-amylase and α-glucosidase activities, suggesting potential for postprandial glycemic control. (2) Neuroprotective—Tagne et al. [73] found that canistel fruit powder and ethanolic extract exerted neuroprotective effects in an aluminum-induced Alzheimer’s disease rat model, improving cognitive function and increasing brain tissue levels of total protein, acetylcholinesterase, catalase, and GSH while reducing MDA content. (3) Antitumor—Pramod et al. [74] reported that ethanolic bark extract exhibited significant cytotoxicity against HeLa cervical cancer cells, inducing apoptosis and G0/G1 cell cycle arrest via upregulation of p21 (WAF1/CIP1) protein expression. (4) Antimicrobial—Ma et al. [45] demonstrated that canistel seed essential oil inhibited common pathogenic bacteria (Staphylococcus aureus, Escherichia coli, and Bacillus subtilis), with inhibition zone diameters ranging from 11.03 to 16.23 mm. (5) Safety—Sánchez-Recillas et al. [75] conducted an acute toxicity evaluation of a canistel-containing herbal formulation, observing no mortality or significant behavioral abnormalities in rats at doses up to 2000 mg/kg, indicating a favorable safety profile at conventional doses. The single acute toxicity study is insufficient for regulatory approval; subchronic and genotoxicity assessments are critical prerequisites before any human intervention trials can be ethically justified.

Across the pharmacological studies reviewed above, a recurring limitation is the predominance of in vitro and acute in vivo models, with a conspicuous absence of chronic toxicity data and human clinical trials. While the antioxidant and anti-inflammatory activities of canistel extracts are consistently reported across multiple independent laboratories [46,54,66,67,68], three critical gaps undermine the translational potential of these findings. First, the active compounds responsible for the observed bioactivities have rarely been purified to homogeneity and tested individually; most studies use crude solvent extracts, making it difficult to attribute effects to specific molecules. Second, the pharmacokinetic properties—absorption, distribution, metabolism, and excretion—of canistel-derived bioactive compounds remain entirely uncharacterized. Third, the effective concentrations reported in vitro (e.g., IC_50_ = 0.5 μg/mL for seed extract [54]) are orders of magnitude higher than what would be achieved through dietary consumption, raising questions about the physiological relevance of these findings. Until these gaps are addressed through rigorously designed pharmacokinetic and dose–response studies in relevant animal models and, ultimately, in humans, the therapeutic claims for canistel should be interpreted with considerable caution. The biological activities and pharmacological effects of different tissues of canistel are shown in Table 2.

## 7. Food Processing and Comprehensive Utilization

### 7.1. Starch Resource Development

Canistel seeds represent a promising non-conventional starch source, with starch content reaching up to 68.1% on a dry weight basis—substantially higher than many conventional starches. Li et al. [76] characterized isolated seed starch, reporting an amylose content of 33.65% and an A-type crystalline structure. Its crystallinity, gelatinization temperature, and enthalpy values exceed those of maize, wheat, rice, and cassava starches, indicating excellent thermal stability and shear resistance—desirable properties for industrial food applications requiring high-temperature processing [77,78].

He et al. [79] reported that vacuum drying pretreatment facilitated the formation of double-helix structures in starch granules, whereas hot-air drying and freeze-drying disrupted ordered structures, leading to altered physicochemical properties. These findings provide practical guidance for selecting appropriate drying pretreatments during starch isolation.

Luo et al. [80] comprehensively investigated the long-term retrogradation mechanism of canistel seed starch (*P. campechiana* seed starch, PCSS; 0–21 days), finding that the crystal structure transforms from A-type to B+V-type with increasing retrogradation time. The retrogradation rate constant (k) of PCSS was higher than that of rice, wheat, and corn starches, indicating a greater tendency for molecular chain rearrangement into ordered structures—a property that must be managed in product formulations to avoid undesirable textural changes.

Critically, Agama-Acevedo et al. [81] found that starches isolated from unripe canistel pulp and seeds retained high proportions of resistant starch after cooking (approximately 33.3% and 32.6%, respectively). Resistant starch resists small intestinal digestion, functioning as a prebiotic with potential for modulating gut microbiota and improving glycemic response.

Collectively, the starch characterization studies reveal a consistent picture: canistel seed starch is unusually high in amylose, exhibits A-type crystallinity, and retains high proportions of resistant starch after cooking. These properties are rare among underutilized fruit crops and position canistel starch as a potential alternative to high-amylose maize starch for low-glycemic and gut-health applications. However, the practical translation of these findings faces two major hurdles. First, the extraction yield and purity of starch from seeds are highly dependent on drying pretreatment, and an economically viable, scalable isolation protocol has not yet been developed. Second, the techno-functional properties—such as pasting behavior, gelation capacity, and freeze–thaw stability—remain inadequately characterized for real-world food formulation contexts. Future research should prioritize pilot-scale extraction optimization and comprehensive rheological characterization to bridge the gap between fundamental starch science and industrial application.

### 7.2. Polysaccharide Extraction and Bioactivity

Beyond starch, canistel seed polysaccharides represent another important class of bioactive macromolecules. Using response surface methodology, Ma et al. [82] optimized ultrasound-assisted extraction, identifying optimal conditions—ultrasonic temperature 79 °C, extraction time 69 min, and liquid-to-material ratio 41 mL/g—achieving a polysaccharide yield of 15.94%. Following purification, a homogeneous polysaccharide fraction (PCSP-1, molecular weight 67.9 kDa) was obtained, with monosaccharide composition predominantly glucose (86.65%) and mannose (4.62%). PCSP-1 exhibited strong concentration-dependent scavenging activities against DPPH, ABTS, hydroxyl, and superoxide anion radicals, with IC_50_ values of 10.31, 29.9, 98.72, and 358.22 μg/mL, respectively—comparable to those of many well-studied plant polysaccharides. These values are comparable to those of established plant polysaccharides such as those from Panax ginseng and Astragalus membranaceus, but in vivo efficacy data for PCSP-1 are yet to be generated. The promising in vitro antioxidant activity warrants further investigation in relevant animal models of oxidative stress.

### 7.3. Food Product Development

Canistel pulp is being progressively incorporated into diversified food products. Kariyawasam et al. [83] developed a novel fruit-based beverage by blending canistel pulp with coconut milk and employing α-amylase enzymatic hydrolysis to effectively improve the starchy mouthfeel, resulting in a product rich in lauric acid, potassium, and calcium. Anjali et al. [84] found that canistel pulp powder, as a natural colorant and antioxidant source, enhanced the color, flavor, and textural properties of ice cream. Anjo et al. [85] added freeze-dried canistel pulp as a natural food dye, demonstrating photostability suitable for food applications—an important consideration given the growing consumer preference for “clean-label” natural colorants.

### 7.4. Carotenoid Stabilization: Encapsulation and Oil-Based Fortification

The high carotenoid content of canistel offers substantial nutritional and colorant potential, yet carotenoids are inherently susceptible to degradation by light, heat, and oxygen. Two distinct technological approaches have been investigated to address this challenge [86,87]: (1) Liposome encapsulation—Xiao [59] employed response surface methodology to optimize liposome encapsulation of canistel carotenoids (optimal conditions: cholesterol 20 mg, lecithin 80 mg, aqueous-to-lipid phase volume ratio 3:1). Encapsulation efficiencies reached 95.12% for lycopene and 94.87% for β-carotene. Stability studies demonstrated that after 15 days of storage at 4 °C and 27 °C in the dark, as well as after heating at 30–80 °C for 50 min, the retention rates of liposome-encapsulated carotenoids were significantly higher than those of unencapsulated extracts. Furthermore, in vitro simulated digestion confirmed that encapsulated carotenoids exhibited significantly higher micellization efficiency, with bioaccessibility enhanced by more than 10%. (2) Oil-based fortification—Jans et al. [88] developed a solvent-free green process for β-carotene-fortified coconut oil using canistel as the carotenoid source. Using response surface methodology with a central composite design, they investigated the effects of oil ratio (50–100%, *w*/*v*) and heating temperature (30–60 °C). The optimal conditions (52.4 °C, 50% oil ratio) achieved a predicted β-carotene concentration of 2.22 μg/mL. Fortified oil demonstrated significantly superior nutritional indices compared to commercial coconut oil: total phenolic content 17.37 ± 2.04 mg GAE/g, total flavonoid content 11.33 ± 0.89 mg QE/g, and DPPH antioxidant activity 9.78 ± 0.53 mg TE/g. Importantly, oxidative stability was significantly enhanced, evidenced by lower peroxide value (0.05 ± 0.01 meq O_2_/kg) and acid value (0.10 ± 0.01 mg KOH/g), confirming that β-carotene serves dual functions as both a nutrient fortifier and an antioxidant synergist.

These two approaches offer complementary pathways: liposome encapsulation is well-suited for high-purity functional delivery systems, whereas oil-based fortification aligns with daily edible oil consumption scenarios, offering operational simplicity, low cost, and solvent-free processing, demonstrating strong potential for “clean-label” functional food development.

## 8. Challenges and Future Perspectives

### 8.1. Critical Research Gaps

Compared with major tropical fruits such as mango and avocado, the body of canistel research is substantially smaller, with most studies published only within the past decade. (1) Insufficient germplasm characterization: Current collection efforts in China remain geographically limited (primarily Guangxi and Yunnan). Systematic introduction and evaluation of elite germplasm from the species’ Mesoamerican center of origin have not been undertaken, limiting the genetic base available for breeding [27]. Moreover, comprehensive genetic diversity assessments using high-resolution molecular markers are lacking for most accessions. (2) Limited cultivar diversity: Only three improved cultivars (‘Xiantao 1’, ‘Xiantao 2’, and ‘Yunre-205’) have been formally released. All share common limitations—large seed size, low edible rate (≤70%), and inconsistent fruit size—and there are no purpose-bred cultivars specifically tailored for distinct end-uses such as fresh consumption, processing, or functional ingredient production [28,29]. (3) Weak molecular and genomic research: While the chloroplast and nuclear genomes have been sequenced, functional genomics (e.g., gene cloning, expression regulation, metabolic pathway elucidation) remains virtually unexplored. The biosynthetic pathways underlying key metabolites—carotenoids, phenolic compounds, and triterpenoids—are entirely uncharacterized at the molecular level, severely constraining targeted breeding and synthetic biology approaches [27]. (4) Narrow product portfolio and low industrialization: Canistel processing remains predominantly at the primary level (fresh consumption, simple jams, and beverages). High-value functional products (e.g., encapsulated carotenoids, resistant starch isolates, bioactive polysaccharides) are scientifically promising but commercially underdeveloped [44]. (5) Inadequate toxicological and clinical validation: Acute toxicity has been assessed in only one study [75], while chronic toxicity, genotoxicity, teratogenicity, and systematic pharmacokinetic studies are entirely absent. Without these data, the development of canistel-based functional foods or pharmaceuticals will face regulatory barriers.

### 8.2. Future Research Priorities

To accelerate the scientific understanding and industrial utilization of canistel, we propose the following priority directions: (1) Germplasm enhancement and pre-breeding—Systematic exploration and collection of wild and landrace germplasm from Mexico, Central America, and the Caribbean, coupled with genotyping-by-sequencing or whole-genome resequencing, would establish a robust foundation for association mapping of key traits (fruit size, seed number, edible rate, stress tolerance). International collaboration and germplasm exchange agreements should be actively pursued. (2) Functional genomics and metabolomics—Integration of transcriptomics, metabolomics, and genome-wide association studies could elucidate the regulatory networks governing biosynthesis of carotenoids, phenolic compounds, and starch. Key candidate genes (e.g., phytoene synthase, carotenoid cleavage dioxygenases, starch synthases) should be functionally validated and leveraged for marker-assisted selection or gene editing. (3) Postharvest physiology and shelf-life extension—Current storage life (~12 days at ambient) is insufficient for commercial distribution. A systematic factorial study should be designed to test the combined effects of 1-methylcyclopropene (1-MCP) treatment, modified atmosphere packaging, and temperature management. The optimal combination should then be validated at pilot scale (≥100 kg fruit) to assess economic feasibility and consumer acceptability [43]. (4) Value-added processing innovation—We recommend that future research prioritize the development of a pilot-scale starch isolation process that maintains the high resistant starch content (>30%) while achieving economically viable yields (>60% recovery). For carotenoid encapsulation, the transition from liposome-based systems (which are costly and difficult to scale) to food-grade Pickering emulsions or spray-dried microcapsules should be explored as more commercially feasible alternatives. (5) Comprehensive safety evaluation—Regulatory approval of canistel-derived functional ingredients will require subchronic and chronic oral toxicity studies, genotoxicity assays (Ames test, micronucleus test), and, ultimately, human clinical trials evaluating bioavailability, pharmacokinetics, and efficacy for specific health claims (e.g., glycemic management, anti-inflammation).

## 9. Conclusions

Canistel is a multipurpose tropical fruit tree with significant nutritional, phytochemical, and pharmacological potential, yet its global development remains in its infancy. This review has synthesized current knowledge across botany, germplasm resources, propagation and cultivation, nutritional and phytochemical composition, pharmacological activities, and food processing. The species exhibits notable genetic diversity in its center of origin, but this diversity is under threat; meanwhile, Chinese breeding programs have produced improved cultivars adapted to local conditions. Propagation and orchard management practices have been established, and the fruit and its by-products show promise as sources of carotenoids, phenolics, and high-amylose resistant starch. Preclinical evidence supports a range of bioactivities, but translational gaps remain, including insufficient clinical and toxicological data. Moving forward, an integrated strategy combining international germplasm exchange, functional genomics, green processing technologies, and clinical validation is essential. With such efforts, canistel can transition from an underutilized local fruit to a globally recognized functional food and nutraceutical resource.

## 10. Literature Search Strategy

A systematic literature search was conducted in November 2025 using the following databases: Web of Science, Scopus, ScienceDirect, PubMed, SpringerLink, and China National Knowledge Infrastructure (CNKI). The search covered the period from 1990 to 2025. The following search terms were used in combination: “*Pouteria campechiana*”, “canistel”, “yellow sapote”, “egg fruit”, “germplasm”, “breeding”, “cultivation”, “propagation”, “phytochemistry”, “carotenoids”, “phenolic compounds”, “starch”, “polysaccharides”, “antioxidant”, “anti-inflammatory”, “hepatoprotective”, “food processing”, and “encapsulation”. Only peer-reviewed articles, book chapters, and conference proceedings with verifiable information were included. Duplicate reports and studies without methodological details were excluded. A total of 86 references were selected for this review.

## Figures and Tables

**Figure 1:**
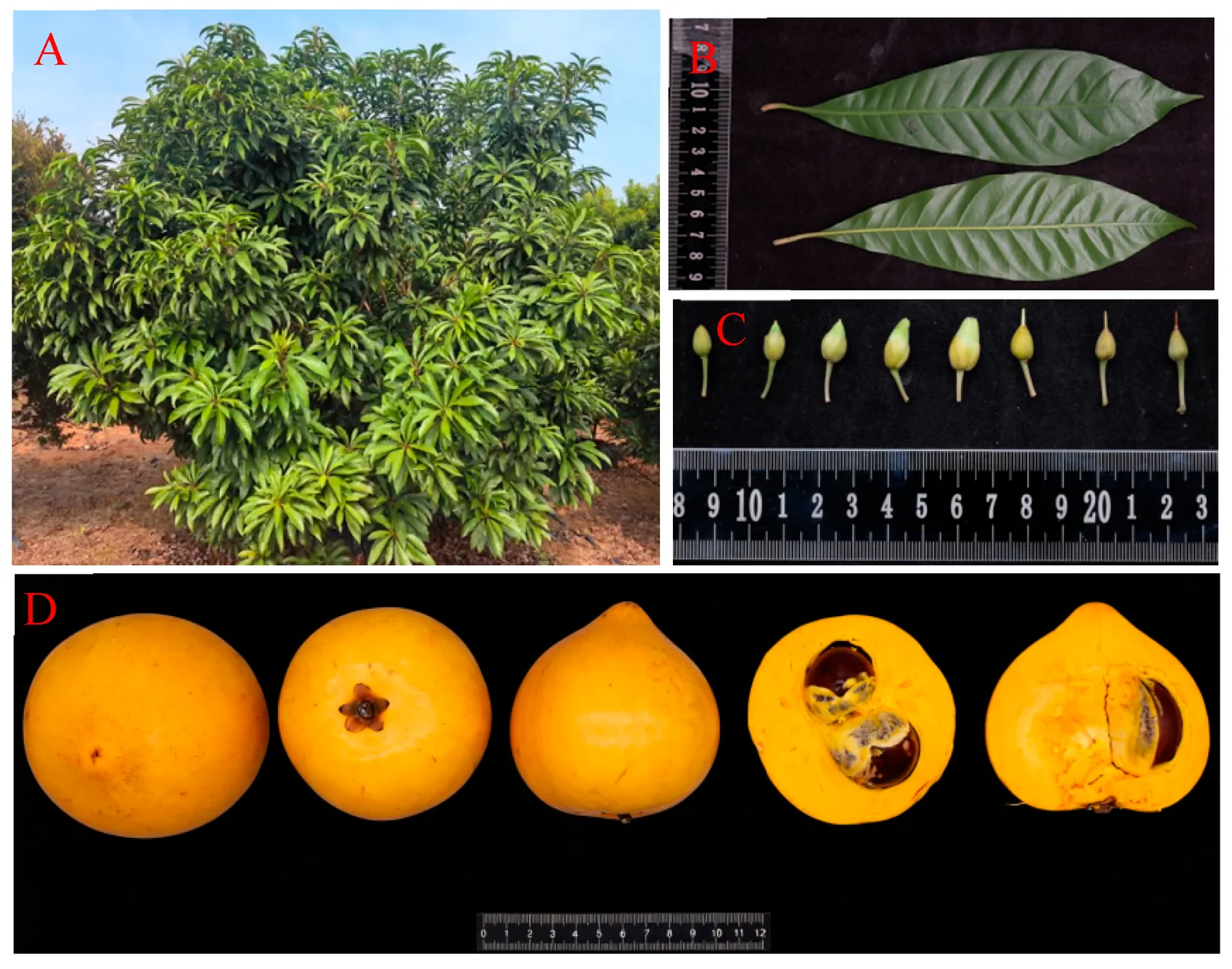
Characteristics of tree morphology, leaves, floral characteristics, fruits, and seeds of the canistel cultivar ‘Xiantao 1’: (**A**) Round-headed canopy; (**B**) Oblanceolate leaf; (**C**) Flower buds at different flowering stages; (**D**) Mature fruits with egg-yolk-yellow flesh and seeds.

**Figure 2:**
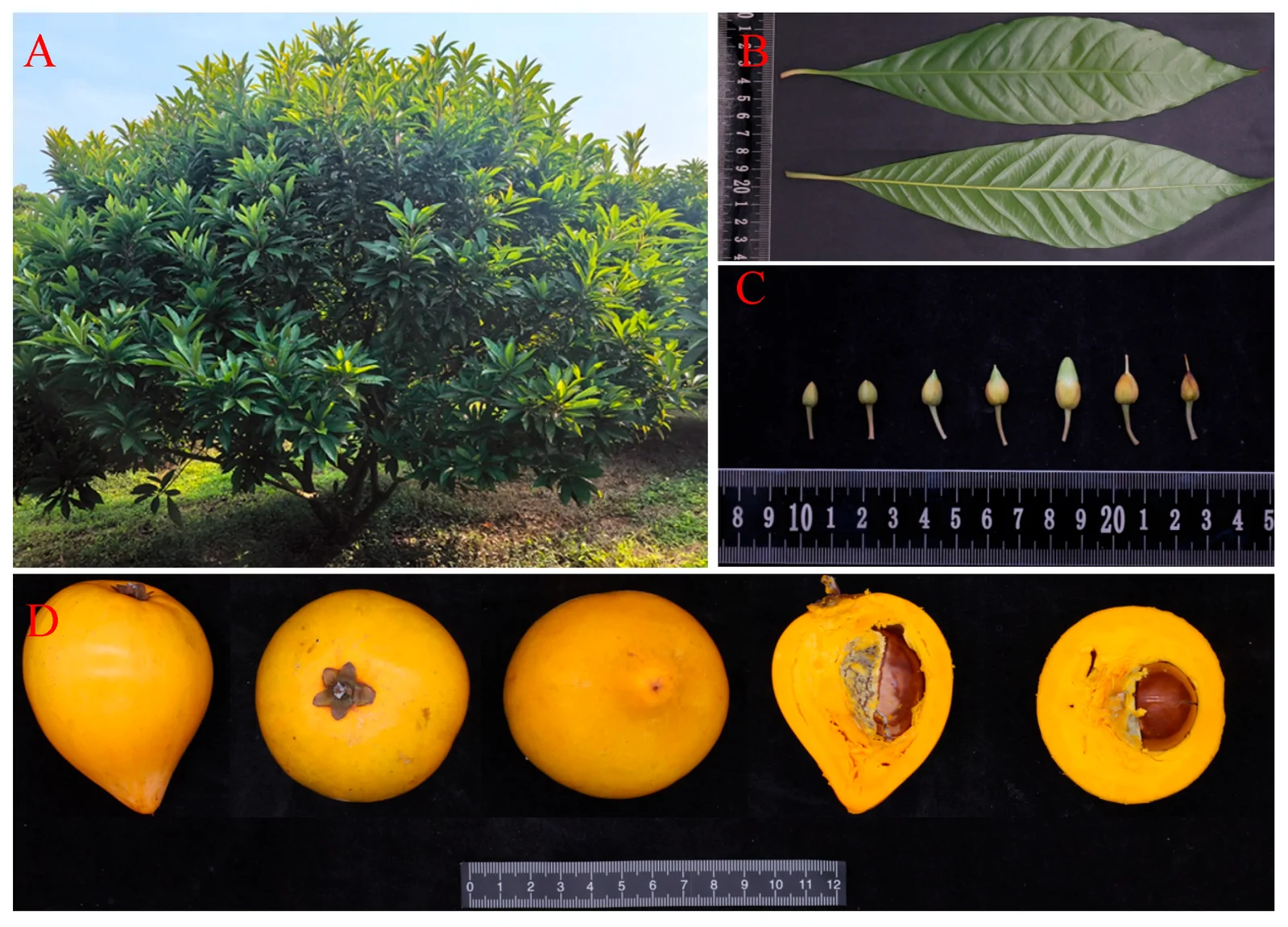
Characteristics of tree morphology, leaves, floral characteristics, fruits, and seeds of the canistel cultivar ‘Xiantao 2’: (**A**) Round-headed canopy; (**B**) Oblanceolate leaf; (**C**) Flower buds at different flowering stages; (**D**) Mature fruits.

**Figure 3:**
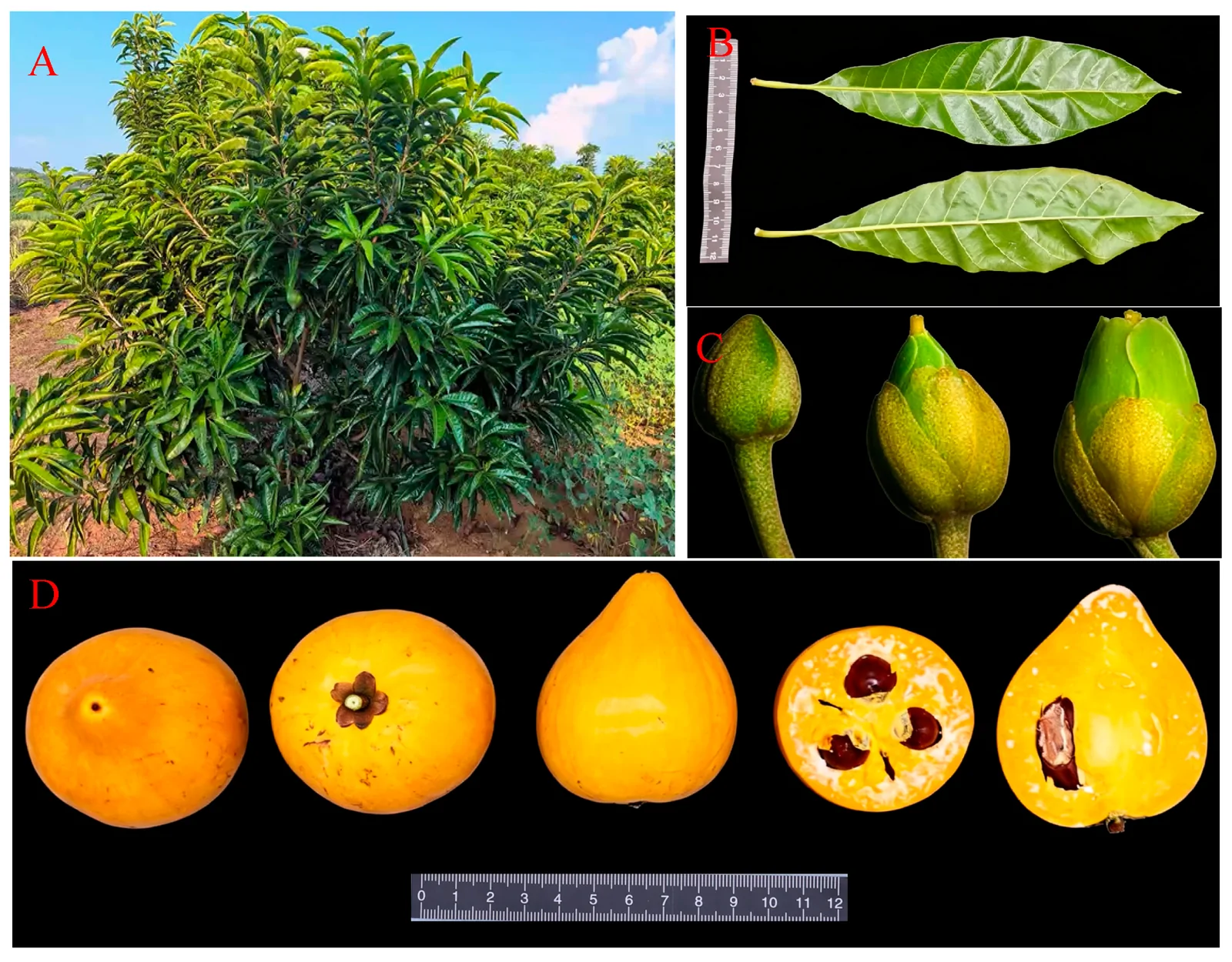
Characteristics of tree morphology, leaves, floral characteristics, fruits, and seeds of the canistel cultivar ‘Yunre-205’: (**A**) Mature tree; (**B**) Oblanceolate leaf with rugose mesophyll; (**C**) Flower buds at different flowering stages (scale bar = 200 μm); (**D**) Mature fruits.

**Table 1:** The major economic traits of representative canistel cultivars from different regions.

Cultivar	Origin	Average Fruit Weight (g)	Flesh Texture	Edible Rate (%)	Key Features	Reference
‘Xiantao 1’	China (Guangxi)	233.7	Dry, mealy	70.1	Peach-shaped; early bearing; high yield	[26]
‘Xiantao 2’	China (Guangxi)	158.5	Dry, mealy	73.9	slightly later-maturing than ‘Xiantao 1’	[27]
‘Yunre-205’	China (Yunnan)	210.7	Dry, mealy	85.8	High yield	[23,28,29]
‘Bruce’	USA (Florida)	510~680	Moist	Not reported	Excellent flavor, color	[24]
‘Fairchild 2’	USA (Florida)	198~369	Moist	Not reported	Good flavor, poor color	[24]
‘Keisau’	USA (Florida)	227~283	Slightly moist	Not reported	Excellent flavor, color	[24]
‘Oro’	USA (Florida)	340~454	Slightly moist	Not reported	Excellent flavor, color	[24]
‘Trompo’	USA (Florida)	255~425	Slightly moist	Not reported	Good flavor, color	[24]
Mexican landraces	Mexico (Yucatán)	180~330	Dry	Not reported	High content of total soluble solids, up to 26~33%	[25]

**Table 2:** The summary of major biological activities and pharmacological effects of different tissues of canistel.

Tissues	Extract/Compound	Key Findings	Reference
Fruit	Ethyl acetate fraction	Identified gallic acid, (+)-gallocatechin, (+)-catechin, myricitrin; DPPH IC_50_ = 146.3 μg/mL	[46]
Fruit	Methanolic extract	Identified two phenolic acids and six flavonoids	[47]
Leaf	Methanolic extract	Isolated six stilbenoids and six flavonoid glycosides	[48]
Seed, leaf	Ethanolic extract	85% inhibition of carrageenan-induced inflammation	[49]
Seed, leaf, peel, pulp	Methanolic extract	Seed extract strongest antioxidant (IC_50_ = 0.5 μg/mL)	[54]
Pulp, seed	Ultrasound-assisted polyphenol extract	Seed polyphenol yield 2.65% vs. pulp 0.74%	[66]
Leaf	Methanolic extract	Stimulated macrophage phagocytosis and NO production	[67]
Fruit	PCP-A polysaccharide	Reduced colitis in mice via p38MAPK/NF-κB inhibition	[68]
Fruit	Aqueous extract	Hepatoprotective at 250 mg/kg comparable to silymarin	[70]
Leaf, fruit	Methanolic extract	Inhibited α-amylase and α-glucosidase	[72]
Fruit	Ethanolic extract	Neuroprotective in Alzheimer’s rat model	[73]
Bark	Ethanolic extract	Cytotoxic to HeLa cells; G0/G1 arrest	[74]

## Data Availability

No new data were created or analysed in this study. Data sharing is not applicable to this article.
